# Low-level night-time light therapy for age-related macular degeneration (ALight): study protocol for a randomized controlled trial

**DOI:** 10.1186/1745-6215-15-246

**Published:** 2014-06-24

**Authors:** Claire McKeague, Tom H Margrain, Clare Bailey, Alison M Binns

**Affiliations:** 1 School of Optometry and Vision Sciences, Cardiff University, Cardiff CF24 4 LU, UK; 2 Bristol Eye Hospital, Lower Maudlin Street, Bristol BS1 2LX, UK; 3 Division of Optometry and Visual Science, School of Health Sciences, City University, London EC1V 0HB, UK

**Keywords:** Age-related macular degeneration, Biomarker, Hypoxia, Light mask, Randomized controlled trial

## Abstract

**Background:**

Age-related macular degeneration (AMD) is the leading cause of blindness among older adults in the developed world. The only treatments currently available, such as ranibizumab injections, are for neovascular AMD, which accounts for only 10 to 15% of people with the condition. Hypoxia has been implicated as one of the primary causes of AMD, and is most acute at night when the retina is most metabolically active. By increasing light levels at night, the metabolic requirements of the retina and hence the hypoxia will be considerably reduced. This trial seeks to determine whether wearing a light mask that emits a dim, green light during the night can prevent the progression of early AMD.

**Methods/design:**

ALight is a Phase I/IIa, multicentre, randomized controlled trial. Sixty participants (55 to 88 years old) with early AMD in one eye and neovascular AMD (nAMD) in the fellow eye will be recruited from nAMD clinics. They will be randomized (in the ratio 1:1), either to receive the intervention or to be in the untreated control group, stratified according to risk of disease progression. An additional 40 participants with healthy retinal appearance, or early AMD only, will be recruited for a baseline cross-sectional analysis. The intervention is an eye mask that emits a dim green light to illuminate the retina through closed eyelids at night. This is designed to reduce the metabolic activity of the retina, thereby reducing the potential risk of hypoxia. Participants will wear the mask every night for 12 months. Ophthalmologists carrying out monthly assessments will be masked to the treatment group, but participants will be aware of their treatment group. The primary outcome measure is the proportion of people who show disease progression during the trial period in the eye with early AMD. A co-primary outcome measure is the rate of retinal adaptation. As this is a trial of a CE-marked device for an off-label indication, a further main aim of this trial is to assess safety of the mask in the cohort of participants with AMD.

**Trial registration:**

International Standard Randomised Controlled Trials Register: ISRCTN82148651

## Background

Age-related macular degeneration (AMD) is the leading cause of blindness in the developed world [1] and is responsible for more than 50% of visual impairment registrations in the UK [2]. For the majority of people with AMD, there is no treatment. The remaining 10 to 15% of people with the advanced, neovascular, form of the disease (nAMD) are mainly treated with intra-ocular injections of ranibizumab, an anti-vascular endothelial growth factor agent [3]. Follow-up treatment for these patients is long-term and places a significant burden on the UK National Health Service. Indeed, advanced AMD currently costs the British economy £1.2 billion to £3.7 billion per annum [2,4,5]. Furthermore, the disease is associated with depression, falls and social isolation [6,7]. Given this significant socioeconomic problem, there is a great need to evaluate potential therapeutic interventions that attempt to treat the disease at an early stage, to prevent vision loss from occurring.

Age-related macular degeneration is characterized by the dysfunction and death of photoreceptors in the central retina. There is an increasing amount of evidence to suggest that hypoxia plays a major role in its pathogenesis [8,9]. This has been attributed to various factors, including a disruption of choroidal circulation [10-13], and thickening and deposition of drusen at Bruch’s membrane [9,14]. The latter increases the distance over which oxygen must travel to reach the retinal pigment epithelium from the choroidal circulation. Disease-related changes to Bruch’s membrane also impair the diffusion of nutrients and growth factors, which consequently exacerbates choroidal perfusion abnormalities, promoting further hypoxia [8,15].

Even in the healthy retina, intraretinal oxygen profiles obtained from animals show that the oxygen tension at the proximal side of the photoreceptor inner segments is close to zero in darkness [16,17]. This is attributable to the metabolic demand of the ‘dark current’ in the retina’s approximately 120 million rods [18]. When this limited supply of oxygen to the outer retina is further compromised by the changes to the choroidal circulation and Bruch’s membrane that occur in AMD, hypoxia may result. This hypoxia could be the precursor for increased vascular endothelial growth factor production and apoptosis [19,20].

The fragile balance between metabolic demand and oxygenation is exemplified functionally by the adverse effects of hypoxia on colour vision [21-23], dark adaptation [24,25], mesopic sensitivity [26] and the electroretinogram [12,27-29]. To date, there is no direct evidence of the effect of hypoxia on visual function in AMD. However, there is evidence that scotopic threshold elevation and prolonged electroretinogram implicit times are associated with areas of reduced choroidal blood flow in AMD [30,31] and pilot data from our laboratory show a transient reduction in scotopic thresholds in an individual with early AMD whilst inhaling oxygen.

Environmental manipulation of light levels can substantially reduce the metabolic demands on the outer retina, thereby reducing the need for oxygen, and potentially providing an intervention that would delay the progression of conditions with a hypoxic aetiology [32]. A pilot study by Arden et al. [33] found no adverse effects from the provision of low-level night lighting over the course of 12 months in individuals with diabetic retinopathy [33]. Furthermore, a recent clinical trial in patients with diabetic macular oedema who wore a low-level light mask during the night for 6 months reported a reduction in oedema and an improvement in functional measures, which was attributed to the obviation of night-time hypoxia [34]. In this trial, we will be using the same intervention (low-level light therapy) to determine its ability to prevent the development of AMD.

## Methods/design

### Trial objectives

The primary aim of this study is to collect preliminary Phase I/IIa proof-of-concept trial data from people with early AMD in one eye and advanced nAMD in the fellow eye, recruited from a hospital nAMD clinic, in order to assess the impact of low-level night-time light therapy, compared with no intervention, on disease progression in the eye with early AMD. A further main aim of this trial is to assess safety of the mask in the cohort of participants with AMD.

The secondary aims of the study are to:

1. Establish the effect of low-level night-time light therapy, compared with no-treatment control, on secondary outcome measures, including: change in drusen volume from baseline in the eye with early AMD; ranibizumab retreatment rates in the fellow eye with nAMD; progression of early AMD on the basis of change in functional outcome measures; change in health-related quality of life (assessed using the EuroQol EQ-5D instrument); change in self-reported visual function assessed using the 48-item Veterans Affairs Low-Vision Visual Functioning Questionnaire (VA LV VFQ-48).

2. Establish the acceptability of low-level night-time light therapy in people with AMD by monthly qualitative interviews.

3. Determine the effect of low-level night-time light therapy on sleep patterns by conducting the Pittsburgh Sleep Quality Index (PSQI) questionnaire every month with both intervention arms by interview with the study investigator.

4. Establish the relationship between baseline functional biomarker outcomes and the severity of AMD (assessed using the simplified Age-Related Eye Disease Scale (AREDS) and initial drusen volume).

5. Evaluate the ability of all clinical tests to act as prognostic biomarkers for AMD progression.

6. Evaluate the ability of all clinical tests to act as predictive biomarkers for low-level night-time light therapy in people with AMD.

7. Compare the sensitivity of all clinical tests to disease progression over 12 months.

### Study design and setting

ALight is a Phase I/IIa prospective proof-of-concept randomized controlled trial, consisting of two parallel groups. Trial recruitment and data collection will take place at the Medical Retina Clinic, Bristol Eye Hospital. Additional cross-sectional data will be collected from people with healthy eyes, or only early signs of AMD, at the Cardiff University School of Optometry and Vision Sciences.

### Eligibility criteria

#### ***Inclusion criteria***

• Between the ages of 55 and 88 years.

• Early Treatment of Diabetic Retinopathy Study (ETDRS) visual acuity of 0.3 logMAR (Snellen 20/40 or 6/12) or better in the test eye.

• Early AMD in the study eye.

• nAMD in the fellow eye, within a month of the third Ranibizumab injection (trial only).

• Willing to adhere to allocated treatment for duration of trial.

#### ***Exclusion criteria***

• Ocular pathology other than macular disease.

• Significant systemic disease or medication known to affect visual function.

• Systemic disease that would compromise participation in a 1-year study (trial only).

• Insufficient English language comprehension.

• Cognitive impairment as determined using an abridged Mini Mental State Examination [7].

• Oxygen mask worn at night.

#### ***Suspension criteria for the trial***

• Participant wishes to discontinue the study.

• Serious adverse events (for example, conversion to nAMD in the test eye) or unexpected changes in clinical status.

### Interventions

Participants allocated to the treatment group will be given a 12-weekly disposable light mask (Polyphotonix Medical, UK) that presents organic light-emitting diode illumination (peak output 502 nm) to both eyes, to be used overnight for 12 weeks. Light masks will be replaced every 12 weeks at the participant’s routine appointment at the nAMD clinic so that the total duration of mask usage is 12 months.

The mask provides a luminance of 75 photopic cd/m^2^. When adjusted for the spectral sensitivity of rod photoreceptors, this equates to 186 scotopic cd/m^2^ [35]. Light transmission by the human eyelid has been found to range between 0.3% and 2% for light in the region of 500 to 505 nm [36-38]. Under these conditions, the pupil diameter in people in the age group 60 to 85 years is approximately 4 mm [39]. This will result in a retinal illuminance in the order of 23 scotopic Td (assuming an eyelid transmission of 1%).

The masks will be pre-programmed to function only between specific hours, that is, 8 pm to 8 am, to prevent misuse. Outside of these hours, they will not illuminate if worn. The mask is activated when a touch sensor on the device is gently covered with a finger for 3 seconds. It will deactivate if not worn continuously for the first 15 minutes, and after that, the light will remain on for the remainder of the 8-hour period.

Treatment acceptability will be evaluated during a monthly interview with the study investigator. Compliance data will be obtained at the monthly visit from the treatment group both (i) through evaluation of a diary of mask usage, and (ii) objectively through data collected on a chip in the mask itself (based on a sensor which logs when the mask is in contact with the face). This provides precise data on the hours the mask is worn each night by each participant. As each mask is programmed with the unique participant identification code, compliance data stored on-chip will be non-identifiable except via the password-protected electronic database.

The current management of patients with early AMD involves advising on lifestyle factors, such as stopping smoking and improving diet. The only other intervention that is based on evidence from a robust randomized controlled trial is the provision of a nutritional supplement consisting of high-dose antioxidants plus zinc. This AREDS group formula (vitamin C, 500 mg; vitamin E, 400 IU; beta carotene, 15 mg and zinc, 80 mg) has been shown to reduce risk of progression from early to advanced AMD by around 20% over 5 years in people with specific features of AMD [40]. However, a recent systematic review by the Cochrane Collaboration indicated that there may be an increased risk of mortality in individuals taking vitamin E and beta carotene supplementation [41]. As this is a recently published document, its advice is not reflected in the current guidelines for the management of AMD. For this reason, the low-level light therapy will be compared with a no-treatment control, rather than with the AREDS group formula as the best current intervention.

Participants in both groups will receive routine ophthalmological care for the eye with nAMD (that is, ranibizumab injections). If the eye with early AMD converts to nAMD, the participant will proceed to ranibizumab treatment for this eye also, and will be withdrawn from the study.

### Outcome measures

This study includes two co-primary outcome measures, reflecting structural status and functional status.

1. The proportion of people who show ‘disease progression’ in the eye with early AMD during the 12 months of the study based on an increase in drusen volume beyond test-retest repeatability limits or a progression to advanced AMD. Software available for the Cirrus optical coherence tomography (OCT) system allows automated assessment of drusen volume. Irrespective of initial drusen volume, approximately 50% of people with AMD show a significant increase in drusen volume over the 12-month study period, that is, beyond test-retest 95% confidence intervals [42]. Conversion to advanced disease will also be considered to be an indicator of disease progression in this analysis. Data from a trial on a similar group indicates that about 10% of people meeting our inclusion criteria will develop advanced AMD within 12 months [43]. Hence, 60% of participants might be expected to show progression based on increased drusen volume or progression to late AMD. The development of advanced AMD will be determined on the basis of ophthalmologist diagnosis at the monthly follow-up appointments at the nAMD clinic.

2. The rate of retinal adaptation (time taken for photoreceptors to recover their sensitivity after being exposed to a bright adapting light).

Secondary outcome measures include: the change in drusen volume over the 12 month follow-up period; the number of ranibizumab retreatments required during the year in the fellow eye with nAMD (assessed through review of medical records at the end of 12 months); changes in visual function including chromatic thresholds, visual acuity and psychophysical 14 Hz flicker thresholds; self-report outcome measures, including health-related quality of life (EQ-5D) and visual function (VFQ-48) [44]; a sleep quality questionnaire (PSQI) and a semistructured interview (conducted monthly by interview) to determine intervention acceptability.

To obtain detailed information about the time course of any therapeutic action, the primary and the first of the secondary outcome measures (based on drusen volume) will be assessed at baseline and then at monthly intervals (using OCT images obtained at the regular nAMD clinic follow-up appointments).

### Participant timeline

**Figure 1:**
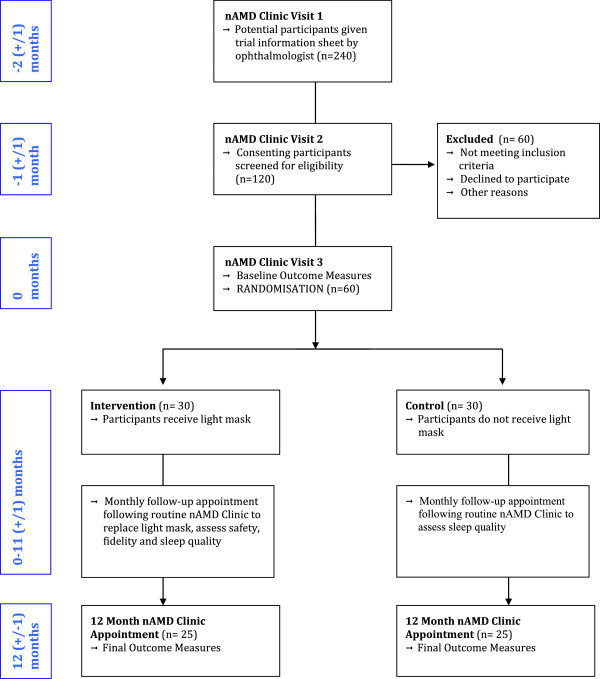
Study flow diagram showing participant timeline.

The study flow diagram (Figure 1) outlines the appointment schedule. In brief, potential participants are identified at their first visit to the nAMD clinic. At the second visit (1 month later), informed consent will be obtained, and screening tests and baseline questionnaires will be carried out. At the third monthly visit, baseline functional tests will be carried out, and participants will be randomly assigned to either the treatment or intervention group. Participants in both intervention groups will attend a short, monthly follow-up appointment following their routine appointment at the nAMD clinic throughout the year. Final outcome data will be collected from control and intervention groups at a 12-month follow-up appointment, scheduled to follow a regular visit to the nAMD clinic.

Participants in the cross-sectional baseline study will attend Cardiff University or the Bristol Eye Hospital for one visit only.

### Recruitment

Sixty participants will be recruited to the trial. Ophthalmologists will identify potential trial participants who are attending for their first appointment at the nAMD clinic at Bristol Eye Hospital, and will provide them with an information sheet and ask for their permission to be contacted by the study investigators. The potential participants will be contacted at least 2 days after their receipt of the information sheet, and invited to meet the study investigator at their next visit to the nAMD clinic (approximately 1 month later) to discuss the trial, provide consent if they choose to participate, and carry out some basic screening tests. Participants will be informed after this screening visit whether they are eligible to take part in the study. Owing to the low number of patients required to participate in the study it is planned for the trial and data collection to take place principally at a single NHS centre, that is, the Bristol Eye Hospital. If needed, additional participants will be recruited from supplementary recruitment centres to ensure that targets are met.

Forty control participants and participants with grade 1 AMD (AREDS simplified scale [45]) for the baseline cross-sectional analysis will be recruited by local optometrists in Bristol and Cardiff, from a database of elderly volunteers, from the Bristol Eye Hospital, from the list of research volunteers at the Cardiff University Eye Clinic and from staff and students of Cardiff University.

### Randomization

Participants will be stratified according to risk of AMD progression (using the AREDS simplified scale [45]), and randomly allocated to receive either the light mask or no intervention in a 1:1 ratio using computer-generated random permuted blocks (both groups will continue to receive ranibizumab injections as required for the fellow eye). The study investigator will be provided with three piles of envelopes by the chief investigator, in order to randomize the participant to either the intervention or control arm of the study. Each pile relates to a different stratum of randomization, that is, grade 2, 3 or 4 of AMD, according to the AREDS simplified scale. The envelopes in each pile will be numbered, and will contain the randomization allocation for each participant.

### Masking

It is not appropriate in this study to use a sham treatment, since a non-illuminated mask may have a physiological effect on the retina and patients would be aware that they weren’t perceiving light and so would be unmasked to their intervention group. The study investigator who collects the outcome data will also be providing the masks and instructions on its usage and, hence, will not be masked to the intervention group. However, the potential for experimenter bias is limited, as the primary outcome measure, disease progression, is an objective measurement carried out by automated computer software. The ophthalmologists who are seeing the participants for their regular ranibizumab injections will be masked, which will prevent any bias in their retreatment decisions for the fellow eye.

### Data collection methods

#### ***Screening visit***

Following a discussion of the study, and the obtaining of informed consent, the study investigator will question the participant regarding ocular and medical history. Retinal photographs will be taken if they have not already been acquired, and repeated if image quality is insufficient to allow evaluation. The OCT and fundus photographs will be assessed to check for eligibility. The Van Herick angle of drainage will be measured, media clarity will be assessed for each eye and the lens graded according to the Lens Opacities Classification System III grading scale [46]. Visual acuity will be assessed in each eye using the ETDRS chart, following a brief refraction, if necessary. An abridged version of the Mini Mental State Examination will be used to assess for cognitive impairment (5 min; [7]).

Those still deemed eligible for inclusion in the study will also complete several questionnaires, through verbal interview with the investigator: visual function (VA LV VFQ-48) [44]; health-related quality of life (EQ-5D); sleep quality (PSQI) [47]; smoking history (pack years); vitamin supplementation; ethnic origin.

#### ***Baseline assessment***

Optical coherence tomography images and fundus photographs obtained during the participant’s routine visit to the ranibizumab clinic will be obtained and analyzed to assess drusen volume and AMD grade, according to the AREDS simplified scale [45]. If the images are of insufficient quality, additional images will be captured.

The ETDRS visual acuity will be assessed in each eye. Chromatic thresholds will be measured for the eye with early AMD using the Colour Assessment and Diagnosis test [48] (City Occupational Ltd). Contrast thresholds to a 14 Hz flickering stimulus will be measured for the eye with early AMD using the procedure outlined by Dimitrov et al. [49]. The rate of parafoveal cone dark adaptation will be determined for the eye with early AMD using a psychophysical procedure described previously [50]. Thresholds will be determined using a psychophysical method, based on a ‘3 down, 1 up’ staircase paradigm [50]. An array of white light-emitting diodes behind a diffusing filter will be used to light-adapt photoreceptors in the central 43.6° of the test eye (the output of the white light-emitting diodes will be modified by a Lee filter, HT015, to provide a retinal illuminance of 5.20 log phot Td.s^-1^, providing a bleach of 85% cone photopigment and 74% rhodopsin). All light levels fall within the safety guidelines set out in British Standard BS EN 15004-2 (2007) [51]. There will be an initial training phase, of around 5 minutes, then the adapting light will be presented, and finally recovery of visual thresholds will be monitored for 25 minutes after exposure to the adapting light.

Those individuals who are assigned to the treatment group will then be given a light mask, and provided with written and oral instructions on its use. There will also be a reprise of the key information provided in the participant information letter about follow-up appointments. Participants will be given written copies to take home of the PSQI questionnaire that will be used in the monthly interview.

#### ***Monthly assessment***

The investigator will access the OCT images and medical records of all participants after they have attended the ranibizumab clinic for each monthly follow-up appointment. This will allow measurement of drusen volume on a monthly basis, which will allow the final analysis to include an assessment of the time course of any therapeutic action. Additionally, reviewing medical records will facilitate monitoring of the conversion rate to advanced AMD in the control and intervention groups in the eye with early AMD at baseline, and of ranibizumab retreatment rates in the eye with nAMD, for adverse event reporting purposes.

Participants in both intervention groups will attend a short, monthly follow-up appointment following their routine ranibizumab clinic appointment, during which both groups will complete the PSQI sleep quality questionnaire. In addition, the treatment group will bring their mask along to each monthly visit to allow objective compliance data (nightly hours of use) to be exported from the device, retraining in mask use if required, functionality of the mask to be checked, masks to be replaced every 12 weeks, and a semistructured interview to be carried out to determine the acceptability of the intervention. These data will be used to address secondary objectives 2 and 3.

#### ***Final (12-month) visit***

This visit will be the same as the baseline visit with the addition of a final semistructured interview assessing the acceptability of the intervention, and the questionnaires (PSQI, VA LV VFQ-48, Euroqol).

#### ***Baseline cross-sectional study***

The 40 participants recruited only to the cross-sectional part of the study will undergo the tests outlined in the baseline assessment visit of the trial.

### Statistical methods

It should be noted that, as a Phase I/IIa proof-of-concept study, this research is designed to assess the acceptability of this therapy for the participants, and to provide preliminary data to support a larger Phase III randomized controlled trial in the future, and so is not powered to detect small effect sizes. To maximize retention, all follow-up visits will be timed to coincide with scheduled appointments at the nAMD clinic. We will aim to recruit 60 people, which, allowing for 15% dropout through the year, should leave a final cohort of 51. This will be sufficient to detect a 50% reduction in people showing progression at a probability level of 0.2, with a power of 80%, and a change in the time constant of cone adaptation of 1 minute to be detected at a probability level of 0.05, with a power of 80%. The additional 40 participants enrolled into the cross-sectional part of the study will allow a total cohort of *n* = 100 to address secondary aim 4.

Analysis will be carried out on an intention-to-treat basis. There will be no interim analysis, but conversions to nAMD in the eye with early AMD at baseline, and ranibizumab retreatments for the fellow eye, will be recorded at each participant visit to the nAMD clinic for safety monitoring purposes.

This trial will primarily be concerned with providing information about the safety of the device in the treatment of AMD, and the magnitude of any treatment effect. On this basis, descriptive statistics will be carried out to summarize the demographic characteristics of the two groups, as well as the proportion of individuals showing disease progression in each group, the magnitude of changes in secondary outcome measures, including drusen volume, measures of visual function, and the self-report tests, and the fellow eye retreatment rates.

The primary outcome measure will be the proportion of patients demonstrating disease progression at 12 months. Comparisons will be performed using stratified Mantel-Haenszel tests and presented as forest plots.

Formal statistical analysis will also include linear regression analysis to investigate changes in drusen volume controlling for intervention arm, baseline drusen volume and patient characteristics (such as age, vitamin supplement intake and history of smoking). Analysis of covariance (ANCOVA) will be carried out to compare the change in secondary outcome measures (for example, drusen volume, functional tests, self-report measures) and the ranibizumab retreatment rates over 12 months between the two intervention arms, controlling for the characteristics listed previously. Note that all analysis except for the ranibizumab retreatment rate pertains to the eye with early AMD. This will address secondary aim 1. To meet our fourth secondary aim, which is to establish the relationship between baseline functional biomarker outcomes and the severity of AMD, we will carry out a one-way analysis of variance (ANOVA) to compare the mean results at baseline between participants with grades of AMD in each group on the AREDS simplified scale. This analysis will take place when the baseline data collection is complete. To assess the ability of the clinical tests to act as prognostic and predictive biomarkers, receiver operating characteristics curves will be constructed to plot the sensitivity and specificity of the baseline measures in predicting outcomes within the control and intervention arms, respectively. This will address secondary aims 5 and 6. When the trial is completed, linear regression analysis will be used to determine how well the change in the functional measures relate to the change in our primary outcome measure (drusen volume), which is a validated biomarker for disease progression. This will address the seventh secondary aim.

### Trial management

The chief investigator has ultimate responsibility for the trial management, assisted by the trial management group. Any important protocol modifications will be communicated to all relevant parties (investigators, research ethics committee, NIHR CRN Portfolio, trial registry, journals, NHS Research and Development, device manufacturer, funding body) by the chief investigator.

A trial steering committee has been established, which will also act as a data monitoring committee. In addition to annual meetings, the committee will be issued with information about any adverse events throughout the course of the study, so that they can decide whether it is appropriate for the study to be terminated. A 3-monthly newsletter will be issued to all investigators and the steering committee, updating on recruitment and other issues, throughout the trial.

### Safety monitoring

All adverse events (AEs) and serious adverse events (SAEs) will be recorded. The chief investigator (AB) will be provided with an update of AEs every month and all SAEs within two working days. This trial does not involve a medicinal product or life-threatening procedure. Hence, the risk of a SAE is low. However, one potential SAE is an increased rate of progression to nAMD in the eye with early AMD at baseline. Although this is at odds with the literature, it is not an impossible outcome. Another potential SAE would be an increased rate of recurrence of nAMD in the fellow eye (resulting in increased ranibizumab retreatment rates). Conversion to nAMD and Ranibizumab retreatment requirement in the fellow eye will be determined by ophthalmologists at the monthly ranibizumab clinic, and recorded by the study investigator through assessment of medical records after each monthly ranibizumab appointment.

Wong et al. [52] carried out a meta-analysis of studies, which had looked at the progression to choroidal neovascularization in the fellow eye when free of advanced disease at study inception. They reported that the cumulative 1-year incidence of nAMD in the 426 patients enrolled in the five studies that evaluated this outcome was 12.2% (confidence interval, 1.7% to 30.6%). Therefore, we have placed an upper limit on the number of people expected to convert to nAMD per month of *n* × (30.6% / 12 months), where *n* is the number of people in the trial who are using the light therapy light mask.

The chief investigator will assess the nature of the AEs and SAEs for seriousness, causality and expectedness. Following the initial report, follow-up data may be requested by the chief investigator. Where the SAE is both related and unexpected, the chief investigator will notify the Trial Steering Committee, the trial sponsor, the National Research Ethics Service North West, and the device manufacturer, who will notify the Medicines and Healthcare Regulatory Agency within 15 days of receiving notification of the SAE.

### Data management

Any trial data will be recorded on spreadsheets using the numerical identifier for each patient. These data will be non-identifiable. All paper records will also use the unique numerical identifier, which will be non-identifiable. The only identifiable personal data will be the paper and electronic copies of the patient database. The paper copy will not be transferred between Cardiff University and the Bristol Eye Hospital, and will be kept in a locked filing cabinet at all times. The electronic database will be stored on a secure Cardiff University computer drive, which is accessible from Bristol and Cardiff via a password-protected connection. The study database will be checked for integrity every month by the chief investigator. At the end of the trial, the data will belong to Cardiff University. At this time, Polyphotonix Medical Ltd (the manufacturer) will have access to the results. Anonymized data will be available for verification at any time by the funding body, the College of Optometrists, on request. Neither the College of Optometrists, nor Polyphotonix Medical Ltd. will influence the data collection or analysis. All data will be kept for 15 years, in line with Cardiff University’s Research Governance Framework Regulations for clinical research.

### Dissemination

A summary of the trial protocol is available to the public through the International Standard Randomised Controlled Trials Register. Any interested individuals may contact the chief investigator for further information. Results will be published in peer-reviewed journals and at International Conferences. The chief investigator has ultimate responsibility for the scientific content of any publications. Dissemination of results to trial participants will take place through a summary-of-findings newsletter sent at the end of the trial to those individuals who indicate a desire to receive an update. Dissemination to the wider community of people with AMD will take place through publication in the Macular Society’s members’ magazine, *Digest*, and through presentations at such events as the Macular Society ‘Top Doctors’ conference. Neither the funding body (the College of Optometrists), the light mask manufacturer (Polyphotonix Medical Ltd), nor the sponsor (Cardiff University) will influence the presentation or the publication of the research.

### Ethical considerations

The study has been approved by the National Research Ethics Service North West, and is registered with the International Standard Randomised Clinical Trials Register. A notice of no objection has been obtained from the Medicines and Healthcare Regulatory Agency. The trial will be conducted in adherence with the Declaration of Helsinki and the Good Clinical Practice guidelines. The chief investigator and the research team will preserve the confidentiality of participants in accordance with the Data Protection Act 1998.

### Audits and inspections

The trial is liable to inspection by the College of Optometrists as the funding organization. The study may also be liable to inspection and audit by Cardiff University under their remit as sponsor.

## Discussion

In this article, we present a clinical trial protocol to evaluate the effect of low-level night-time light therapy in patients with early AMD. To our knowledge, this is the first randomized controlled trial of its kind in AMD. This study will provide the foundation for future large-scale clinical trials. With the prediction of longer life expectancy in the future, the prevalence of AMD and its associated social and economic problems will continue to increase unless a treatment is developed that will stop its progression.

## Trial status

This protocol aims to establish the therapeutic benefit of low-level light therapy on disease progression in AMD. Recruitment will begin in April 2014 and continue until February 2015. Data collection will take place from June 2014 to April 2016.

## Abbreviations

AE: adverse event; AMD: age-related macular degeneration; ANCOVA: analysis of covariance; ANOVA: analysis of variance; AREDS: age-related eye disease scale; CE: Conformité Européenne; ETDRS: Early Treatment of Diabetic Retinopathy Study; nAMD: neovascular age-related macular degeneration; OCT: optical coherence tomography; PSQI: Pittsburgh Sleep Quality Index; SAE: serious adverse event; VALVVFQ-48: 48-item Veterans Affairs Low-Vision Visual Functioning Questionnaire.

## Competing interests

None of the authors has any financial interest in the device, or any other aspect of this trial.

## Authors’ contributions

CM: design, manuscript writing, final approval of the manuscript. TM: conception and design, critical revision and final approval of manuscript. CB: design, critical revision and final approval of manuscript. AB: conception and design, manuscript writing and final approval of the manuscript. All authors read and approved the final manuscript.
